# Uncovering the First Atypical DS-1-like G1P[8] Rotavirus Strains That Circulated during Pre-Rotavirus Vaccine Introduction Era in South Africa

**DOI:** 10.3390/pathogens9050391

**Published:** 2020-05-20

**Authors:** Peter N. Mwangi, Milton T. Mogotsi, Sebotsana P. Rasebotsa, Mapaseka L. Seheri, M. Jeffrey Mphahlele, Valantine N. Ndze, Francis E. Dennis, Khuzwayo C. Jere, Martin M. Nyaga

**Affiliations:** 1 Next Generation Sequencing Unit, Division of Virology, Faculty of Health Sciences, University of the Free State, Bloemfontein 9300, South Africa; 2017219839@ufs4life.ac.za (P.N.M.); tmogotsi16@gmail.com (M.T.M.); RasebotsaS@ufs.ac.za (S.P.R.); 2 Diarrhoeal Pathogens Research Unit, Sefako Makgatho Health Sciences University, Medunsa 0204, Pretoria, South Africa; mapaseka.seheri@smu.ac.za; 3 South African Medical Research Council, 1 Soutpansberg Road, Pretoria 0001, South Africa; Jeffrey.Mphahlele@mrc.ac.za; 4 Faculty of Health Sciences, University of Buea, P.O. Box 63, Buea, Cameroon; valentinengum@yahoo.com; 5 Noguchi Memorial Institute for Medical Research, University of Ghana, P.O. Box LG581, Legon, Ghana; FDennis@noguchi.ug.edu.gh; 6 Centre for Global Vaccine Research, Institute of Infection and Global Health, University of Liverpool, Ronald Ross Building, 8 West Derby Street, Liverpool L69 7BE, UK; Khuzwayo.Jere@liverpool.ac.uk; 7 Malawi-Liverpool-Wellcome Trust Clinical Research Programme, College of Medicine, University of Malawi, Blantyre 312225, Malawi

**Keywords:** atypical strains, genome constellation, reassortment, rotavirus, whole-genome characterization

## Abstract

Emergence of DS-1-like G1P[8] group A rotavirus (RVA) strains during post-rotavirus vaccination period has recently been reported in several countries. This study demonstrates, for the first time, rare atypical DS-1-like G1P[8] RVA strains that circulated in 2008 during pre-vaccine era in South Africa. Rotavirus positive samples were subjected to whole-genome sequencing. Two G1P[8] strains (RVA/Human-wt/ZAF/UFS-NGS-MRC-DPRU1971/2008/G1P[8] and RVA/Human-wt/ZAF/UFS-NGS-MRC-DPRU1973/2008/G1P[8]) possessed a DS-1-like genome constellation background (I2-R2-C2-M2-A2-N2-T2-E2-H2). The outer VP4 and VP7 capsid genes of the two South African G1P[8] strains had the highest nucleotide (amino acid) nt (aa) identities of 99.6–99.9% (99.1–100%) with the VP4 and the VP7 genes of a locally circulating South African strain, RVA/Human-wt/ZAF/MRC-DPRU1039/2008/G1P[8]. All the internal backbone genes (VP1–VP3, VP6, and NSP1-NSP5) had the highest nt (aa) identities with cognate internal genes of another locally circulating South African strain, RVA/Human-wt/ZAF/MRC-DPRU2344/2008/G2P[6]. The two study strains emerged through reassortment mechanism involving locally circulating South African strains, as they were distinctly unrelated to other reported atypical G1P[8] strains. The identification of these G1P[8] double-gene reassortants during the pre-vaccination period strongly supports natural RVA evolutionary mechanisms of the RVA genome. There is a need to maintain long-term whole-genome surveillance to monitor such atypical strains.

## 1. Introduction

Diarrhea persists as a leading infectious mortality cause in children under the age of five worldwide [1]. Group A rotavirus (RVA) is the primary viral etiologic agent for acute gastroenteritis in children under five years of age [2], resulting in annual mortality cases ranging from 122,322 to 215,757 with an estimated 81% reported in sub-Saharan Africa and Southeast Asia [3,4]. To combat RVA diarrhea, especially in countries with high RVA disease burden, the World Health Organization (WHO) recommends incorporation of RVA vaccines into the national immunization programs alongside other childhood vaccines [5]. The WHO has prequalified four vaccines (Rotarix^®^, GlaxoSmithKline, Rixenstart, Belgium; RotaTeq^®^, Merck & Co, USA; ROTAVAC^®^, Bharat Biotech, Hyderabad, India and ROTASIL^®^, Serum Institute of India, Pune, India) for global use [6]. Two vaccines (Rotavin-M1^®^, POLYVAC, Hanoi, Vietnam and Lanzhou lamb rotavirus, Lanzhou Institute of Biological Products, Lanzhou, China) have been approved for national use in Vietnam and China, respectively [7,8]. Human neonatal RVA vaccine (RV3-BB) and bovine human reassortant RVA vaccine candidates as well as neonatal and non-replicating injectable vaccines are in the pipeline [9]. South Africa was the first African country to adopt the monovalent RVA vaccine (Rotarix^®^) in September 2009 into its Expanded Program on Immunization (EPI) (WHO, 2009), which culminated in a 77% reduction in RVA disease during the first year that the vaccine was introduced [10,11].

Rotaviruses belong to the *Reoviridae* family. The RV genome is composed of 11 segments of double-stranded RNA (dsRNA) encapsulated in a three-layered protein capsid. Six structural proteins (VP1–VP4, VP6, and VP7) and five or sometimes six non-structural proteins (NSP1–NSP5/NSP6) that encode the RV genome [2]. The outer capsid proteins, VP7 and VP4, which act as neutralizing agents, are universally applied in the binary classification of RV strains into G and P types, respectively [2]. The contemporary classification of RVA strains is based on whole-genome composition underpinned by the nucleotide homology cutoff values that have been determined for the open reading frame (ORF) of each gene segment [12,13]. The numbers of currently described genotypes are 36 G (VP7), 51 P (VP4), 26 I (VP6), 22 R (VP1), 20 C (VP2), 20 M (VP3), 31 A (NSP1), 22 N (NSP2), 22T (NSP3), 27 E (NSP4), and 22 H (NSP5) (http://rega.kuleuven.be/cev/viralmetagenomics/virus-classification).

The globally predominant RVA genotypes are G1P[8], G2P[4], G3P[8], G4P[8], G9P[8], and G12P[8] [14]. However, RVA strains variability by region is well documented [15]. In Africa, RVA genotypes such as G1P[6], G8P[4], G8P[6], G8P[8], and G9P[6] are substantially prevalent but uncommon elsewhere [14,15,16,17]. Additionally, G3P[8] and G4P[8] genotypes have been on the decline in Africa and have not been detected in many African countries for almost a decade aside from an impromptu emergence of equine-like G3P[6] and G3P[8] in Botswana and Eswatini [18]. RVAs are classified further into three genogroups: Wa-like, which bears a genotype 1 constellation (I1-R1-C1-M1-A1-N1-T1-E1-H1), DS-1-like, which bears genotype 2 constellation (I2-R2-C2-M2-A2-N2-T2-E2-H2), and a relatively minor AU-1-like characterized by genotype 3 constellation (I3-R3-C3-M3-A3-N3-T3-E3-H3) [19]. Typically, G1P[8], G3P[8], G4P[8], G9P[8], and G12P[8] RVA have a Wa-like genotype constellation, whereas G2P[4], G8P[4], and G8P[6] strains usually have a DS-1-like genotype constellation [19]. G1P[8] is the world’s most prevalent genotype accountable for an estimated 50% of RVA infections [20]. The vast antigenic and genetic heterogeneity of G1P[8] strains contributes to the persistent recurrence of VP4 and VP7 protein variants, and the epidemiological fitness of some of these variants might be accountable for their global prevalence [21].

The segmented RNA genome of RVA facilitates reassortment and recombination events, and the error-prone RNA-dependent RNA polymerase promotes high mutation rates [2]. These evolutionary mechanisms lead to the emergence of novel strains and distinct lineages [22]. Intergenogroup reassortment of G1P[8] gene segments has been reported in Africa, Asia, and the Americas [23,24,25,26,27]. These atypical G1P[8] strains were first reported in Okayama Prefecture, Japan during 2012–2013 post-RVA vaccine surveillance of acute gastroenteritis and then in other prefectures, including Aichi, Akita, Kyoto, and Osaka [25,26,27]. Subsequent incidences were then reported during 2013 post-RVA vaccine surveillance in Phetchabun and Sukhothai provinces in Thailand [28,29] and in 2012–2013 during the pre-RVA vaccine period in Hanoi, Vietnam [30]. Although unpublished, sequence data of G1P[8] DS-1-like sequence strains isolated during pre-vaccine period between August–November 2012 in Palawan, Southwestern region of Philippines have been deposited in the GenBank database. Recently, for the first time in the Americas, G1P[8] DS-1-like strains were reported in 2013 during post-RVA vaccination period from the states of Sao Paulo and Goias in Brazil [23]. In Africa, Jere and colleagues reported the emergence of atypical G1P[8] strains during the post-RVA vaccination period in Blantyre, Malawi [24]. It is not definitively resolved whether these atypical G1P[8] strains are widespread. In addition, there is a paucity of information on whole-genome sequences of G1P[8] strains post-vaccine era with only a few countries performing full-genome characterization of the strain [15,21,31,32,33,34,35]. The African Enteric Viruses Genome Initiative (AEVGI) is conducting whole-genome characterization of country-specific pre- and post-vaccine RVA strains in Africa and has identified, for the first time in South Africa, atypical G1P[8] strains that were circulating before vaccine introduction. This study aimed to determine the genetic relationship and the evolutionary origin of these pre-vaccine atypical G1P[8] RVA strains.

## 2. Results

### 2.1. Nucleotide Sequencing

Illumina^®^ MiSeq sequencing yielded 14.7 × 10^5^ reads (379 bp fragment size) and 11.3 × 10^5^ reads (364 bp fragment size) for strains RVA/Human-wt/ZAF/UFS-NGS-MRC-DPRU1971/2008/G1P[8] and RVA/Human-wt/ZAF/UFS-NGS-MRC-DPRU1973/G1P[8], respectively. All the sequences had a phred score of Q ≥ 30 (99.9% base calling accuracy).

### 2.2. Full-Genome Constellation Analysis

Whole-gene sequences of the 11 genes of strains, RVA/Human-wt/ZAF/UFS-NGS-MRC-DPRU1971/G1P[8] and RVA/Human-wt/ZAF/UFS-NGS-MRC-DPRU1973/G1P[8], were determined, and their genotype constellations were revealed as G1-P[8]-I2-R2-C2-M2-A2-N2-T2-E2-H2 (Table 1). The sizes of full-length segments 1 to 11 and their respective open reading frames (ORFs) for the two study strains were determined (Table 1). The ORF sequences for all the 11 genes of these two South African atypical G1P[8] strains were deposited in GenBank under accession numbers MT163245-MT163266.

### 2.3. Sequence and Phylogenetic Analysis

#### 2.3.1. Phylogenetic Analysis of VP7

**Figure 1:**
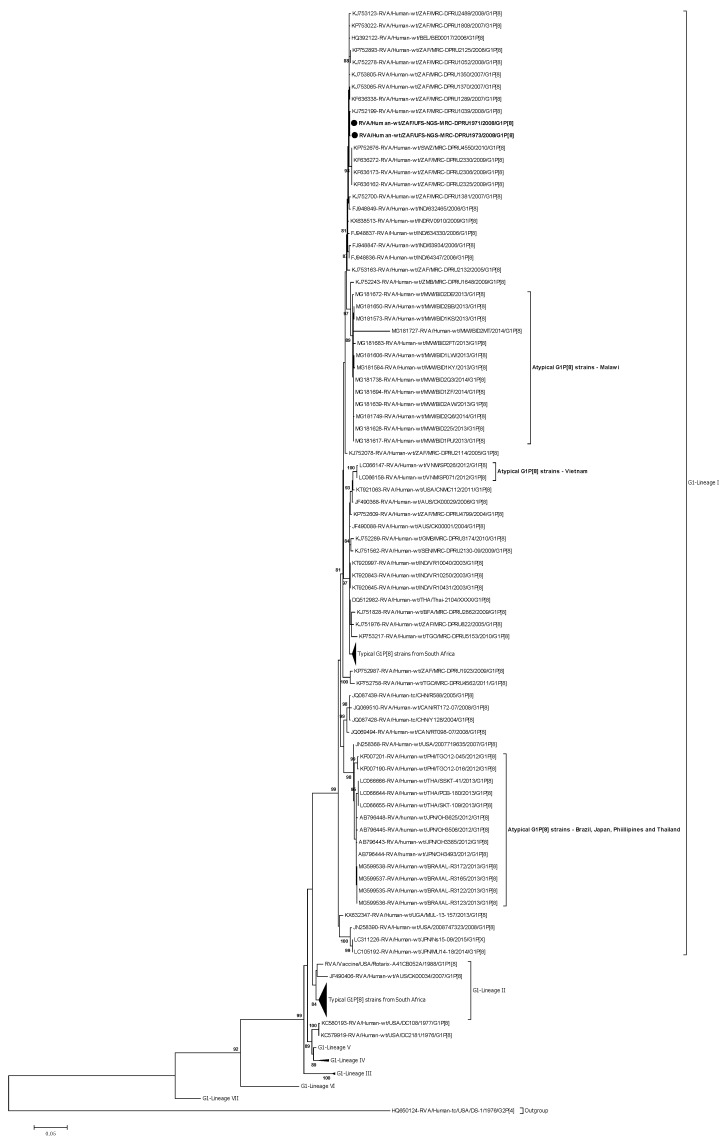
VP7 phylogenetic tree based on the full-length nucleotide sequences. Strains group A rotavirus (RVA)/Human-wt/ZAF/UFS-NGS-MRC-DPRU1971/2008/G1P[8] and RVA/Human-wt/ZAF/UFS-NGS-MRC-DPRU1973/2008/G1P[8] are identified by the black filled circular dots (●). Unusual G1P[8] strains from Malawi, Japan, Thailand, Vietnam, Brazil, and Philippine are indicated. Bootstrap values ≥ 70% are shown adjacent to each branch node. Each scale bar indicates the number of nucleotide substitutions per site.

Phylogenetically, the diversity of the VP7 G1 genes has been established through seven known lineages (I-VII) [36] (Figure 1). The VP7 genes of the atypical G1P[8] study strains, RVA/Human-wt/ZAF/UFS-NGS-MRC-DPRU1971/2008/G1P[8] and RVA/Human-wt/ZAF/UFS-NGS-MRC-DPRU1973/2008/G1P[8], clustered in genetic lineage I, which consisted of a global collection of G1 strains that circulated from 2002 to 2015 (Figure 1). In this lineage I, the two G1 study strains clustered closely together and shared almost absolute gene identities amongst themselves—nt (aa) 99.9% (100%) (Figure 1; Supplementary data 1 (S1)). Analysis of the G1 study strains with locally circulating South African strains retrieved from the GenBank identified the highest sequence identities of 99.8–99.9% (100%) with strain RVA/Human-wt/ZAF/MRC-DPRU1039/2008/G1P8 and clustered closely with this strain that was isolated the same year, 2008, as the two study strains (Figure 1). However, within the same lineage, the VP7 genes of the two G1 study strains from South Africa clustered distinctly away from the atypical G1P[8] strains reported in Brazil, Japan, Malawi, Philippines, Thailand, and Vietnam [22,23,24,25,26] and displayed overall nt (aa) similarities that ranged from 87.0–98.5% (87.1–98.8%) (Table S1 in Supplementary data 2 (S2)). Specifically, the nt (aa) similarities ranged from 96.9–97.0% (98.5%), 96.9–97.1% (98.5–98.8%), 87.0–98%5 (87.1–98.8), 96.5–96.8% (98.2–98.5%), 96.9–97.0% (98.8%), and 97.0–97.1% (96.9%) to the post-vaccination G1 atypical strains reported in Brazil, Japan, Philippines, Malawi, Thailand, and Vietnam, respectively (S1).

When the two South African G1 study strains were compared to the typical G1 strains selected globally, they displayed the highest nt (aa) similarities of 99.7–99.8% (100%) with a European strain, RVA/Human-wt/BEL/BEL00017/2006/G1P[8] (S1). The nt(aa) similarities comparison to representative strains from Africa (Eastern Africa, Southern Africa, and West Africa), America, Asia, Europe, and Oceania ranged from 93.9–97.6% (94.2–98.2%), 97.1–99.5% (98.2–100%), 97.1–97.9% (96.9–98.2%), 93.1.5–97.6% (94.8%–98.5%), 96.4–99.6% (97.8–99.7%), 99.7–99.8% (100%), and 93.7–98.4% (94.5–98.8%), respectively (Table S2 in S2). In addition, comparison of the VP7 genes of the two study strains to cognate gene sequence of the Rotarix^®^ and RotaTeq^®^ RV vaccine strains displayed nt (aa) identities that ranged from 94.2–94.3% (95.7%) and 91.0–91.1% (93.2%), respectively (S1).

##### Analysis of the VP7 Neutralization Epitopes

**Figure 2:**
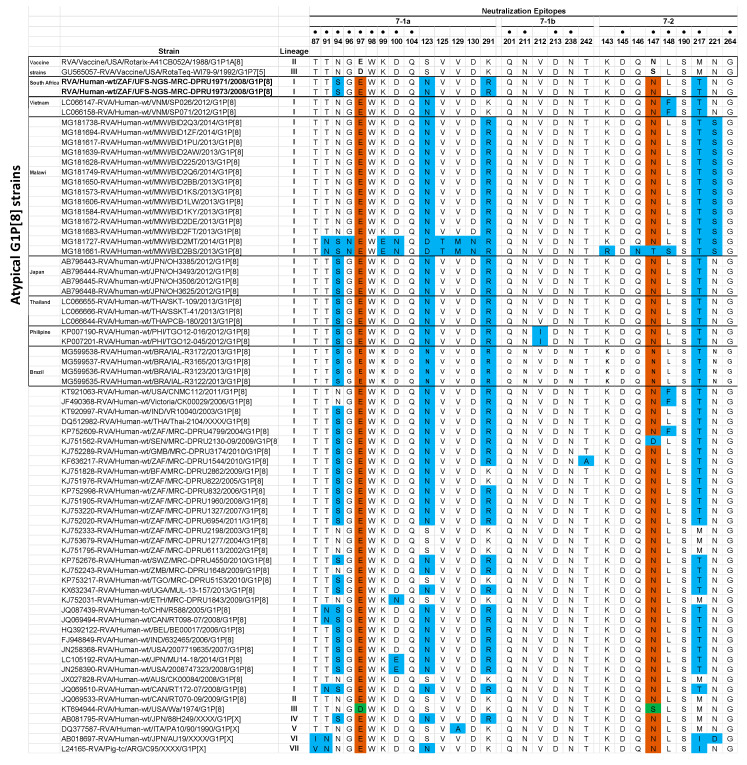
Alignment of antigenic residues in VP7 between the strains contained in Rotarix^®^ and RotaTeq ^®^ and wild type G1 strains. Antigenic residues are divided in three epitopes (7-1a, 7-1b, and 7-2). Amino acids that differ between Rotarix^®^ and RotaTeq^®^ are indicated in boldface. Sky blue colored residues are residues that are different from both Rotarix^®^ and RotaTeq^®^, green colored residues are different from Rotarix^®^, and brown colored residues are different from RotaTeq^®^. Amino acid changes that have been shown to escape neutralization with monoclonal antibodies are indicated with a black dot. Atypical G1P[8] and countries of detection are indicated on the left side of the figure. South Africa atypical G1P[8] strains are in boldface characters.

The VP7 genes contain three established neutralization epitopes: 7-1a, 7-1b, and 7-2. Twenty-nine amino acids (14 residues in 7-1a, 6 residues in 7-1b, and 9 residues in 7-2) define the three VP7 antigenic epitopes [37]. The VP7 neutralization epitope sites of the two South African study strains were aligned and mapped against cognate neutralization sites of the two RV vaccines, Rotarix^®^, and RotaTeq^®^. Four amino acid differences (N94S, S123N, K291R, and M217T) in the VP7 genes of the two South African study strains were identified relative to Rotarix^®^ VP7 neutralization sites, while five amino acid differences (D97E, S123N, K291R, S147N, and M217T) were identified with comparison to RotaTeq^®^ G1 antigenic sites (Figure 2). Antigenically, similar amino acid residues in the VP7 epitopes of the study strains were observed in the corresponding VP7 epitopes of the multiple atypical G1P[8] strains (Figure 2). The VP7 epitopes of the two South African G1 strains were contrasted with those of globally selected lineage I G1 strains. The analysis showed ten amino acid differences (T91N, S94N, D100N, D100E, N123S, R291K, T242A, N147D, L148F, and T217M) (Figure 2).

#### 2.3.2. Phylogenetic analysis of VP4

**Figure 3:**
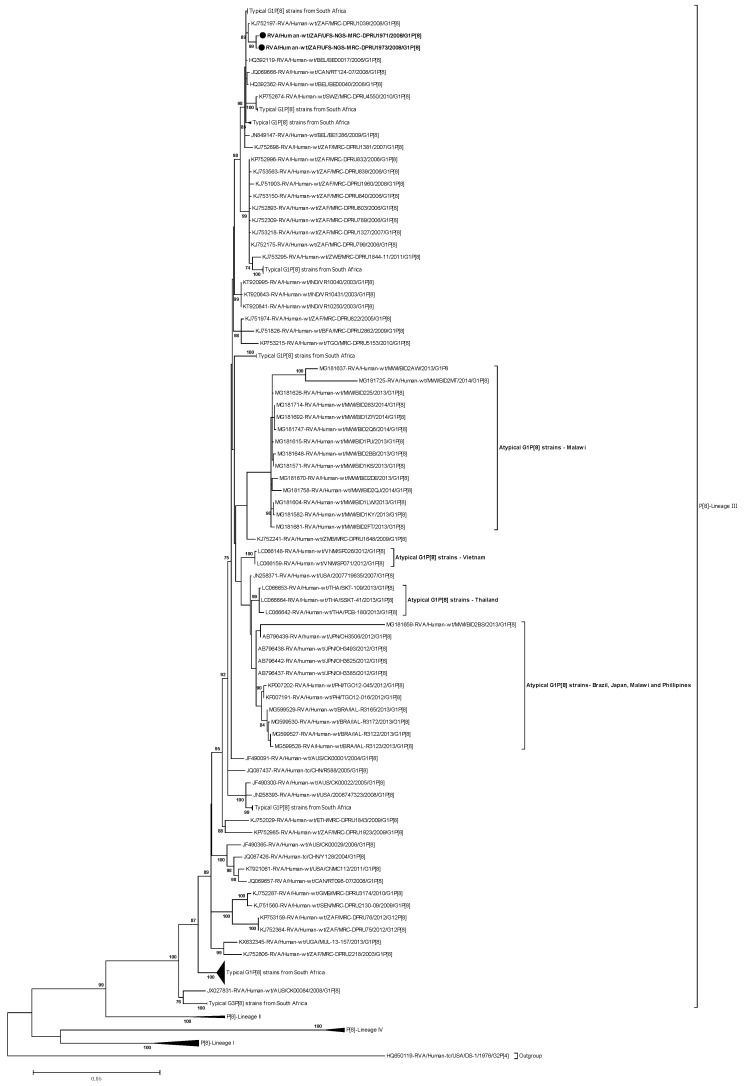
VP4 phylogenetic tree based on the full-length nucleotide sequences. Strains RVA/Human-wt/ZAF/UFS-NGS-MRC-DPRU1971/2008/G1P[8] and RVA/Human-wt/ZAF/UFS-NGS-MRC-DPRU1973/2008/G1P[8] are identified by the black filled circular dots (●). Unusual G1P[8] strains from Malawi, Japan, Thailand, Vietnam, Brazil, and Philippines are indicated. Bootstrap values ≥ 70% are shown adjacent to each branch node. Each scale bar indicates the number of nucleotide substitutions per site.

The VP4 genes of the two atypical G1P[8] study strains were phylogenetically compared to the four established lineages (l-IV) of the P[8] genotypes [38] (Figure 3). The P[8] genes of the South African strains, RVA/Human-wt/ZAF/UFS-NGS-MRC-DPRU1971/2008/G1P[8] and RVA/Human-wt/ZAF/UFS-NGS-MRC-DPRU1973/2008/G1P[8], clustered in lineage III, which consisted of a global collection of P[8] strains that circulated from 2002 to 2014 (Figure 3). Within the P[8]-lineage-III, the two atypical G1P[8] study strains clustered closely together and shared nt (aa) identities of 99.9% (99.7%) amongst themselves (Figure 3; S1). Homology analysis of the P[8] sequences of the two South African strains with sequences of South African strains retrieved from the GenBank demonstrated the highest nt (aa) sequence identities of 99.6% (99.1–99.4%) with strain RVA/Human-wt/ZAF/MRC-DPRU1039/2008/G1P[8] (Figure 3). However, within the same lineage, the VP4 genes of the two atypical strains from South Africa segregated distinctly away from the atypical strains that have been detected in Brazil, Japan, Malawi, Philippines, Thailand, and Vietnam. They exhibited overall nt (aa) similarities that ranged from 95.2–98.0% (95.1–98.6%) (Table S1 in S2). Specifically, the nt (aa) similarities ranged from 97.6–97.8% (98.1–98.6), 98.2–98.5% (98.5%), 95.2–98.0% (95.1–98.2%), 97.7–97.8% (98.1–98.5%), 97.8–98.0% (97.7–98.2%), and 98.1–98.2% (97.9–98.3%) to the post–vaccination atypical strains reported in Brazil, Japan, Malawi, Philippines, Thailand, and Vietnam, respectively (S1). A comparison of the South African P[8] study strains characterized in this study with a global collection of P[8] strains showed their closeness, and the study strains shared the highest nt (aa) similarity of 99.5% (99.1–99.4%) to a Belgian strain, RVA/Human–wt/BEL/BEL00017/2006/G1P[8] (S1). Overall, the nt (aa) similarities in comparison to representative strains from Africa (Eastern Africa, Southern Africa, Western Africa), America, Asia, Europe, and Oceania ranged from 97.4–97.8% (97.8–98.2%), 86.7–99.1% (91.1–99.2%), 86.7–99.1% (91.1–99.2%), 86.6–99.4% (91.0–99.2%), 86.5.–98.9% (91.1–98.7%), 86.8–99.5% (91.2–99.5%), and 86.5–98.5% (91.0%–98.6%), respectively. In addition, the comparison of the atypical VP4 genes to the P[8] genes of the Rotarix^®^ and RotaTeq^®^ vaccine strains displayed nt (aa) identities that ranged from 90.3–90.4% (93.9–94.2%) and 92.3% (95.2%), respectively (S1).

##### Analysis of the VP4 Neutralization Epitopes

**Figure 4:**
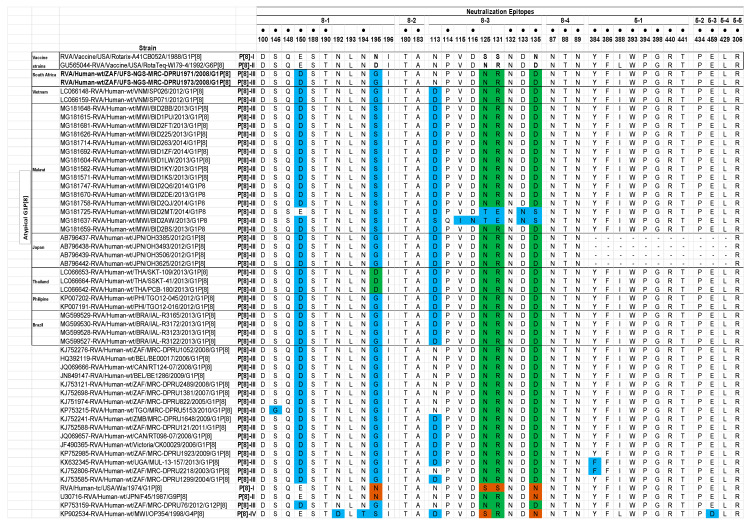
Alignment of antigenic residues in VP4 between the P[8] component of Rotarix^®^ and RotaTeq^®^ vaccines and wild type P[8] strains. Antigenic residues are divided in four antigenic epitopes in VP8* (8-1, 8-2, 8-3, and 8-4) and five antigenic epitopes in VP5* (5-1, 5-2, 5-3, 5-4, and 5-5). Amino acid changes that have been shown to escape neutralization with monoclonal antibodies are indicated with a black dot. Amino acids that differ between Rotarix^®^ and RotaTeq^®^ are indicated in boldface. Green colored residues are residues that are different from Rotarix^®^, brown colored residues are different from RotaTeq^®^, and residues colored in sky blue are different from both Rotarix^®^ and RotaTeq^®^. Dashes (-) indicate no amino acid sequence.

The VP4 spike protein is cleaved by trypsin into two distinct structural proteins, VP8* and VP5* [2]. Analysis of the two South African study strains’ VP4 sequences showed a conserved trypsin cleavage site (arginine) at positions 230, 240, and 581 [39]. Furthermore, the neutralization epitopes in the VP8* and the VP5* regions were analyzed. The VP8* region has four (8-1 to 8-4) neutralization epitopes, while VP5* has five (5-1 to 5-5) (Figure 4) [40]. Comparison of the two South African P[8] strains relative to the Rotarix^®^ and the RotaTeq^®^ P[8] sequences displayed 32 and 35 identical amino acid residues, respectively, spanning the VP4 antigenic epitopes (Figure 4). Amino acid differences between the two P[8] study strains and the P[8] component of vaccine strains were only identified in 8-1, 8-2, and 8-3 VP8* epitopes. Five amino acid differences (E150D, N195G, S125N, S131R, and N135D) were identified in the study strains in relation to Rotarix^®^ P[8] strain, while two amino acid differences (E150D and D195G) were identified relative to P[8] strain of RotaTeq^®^ (Figure 4). Analysis with VP4 epitopes of other atypical G1P[8] strains identified similar amino acid residues with the exception of position 113 in the 8-3 epitope, whereby asparagine was observed in the study strains while other atypical strains had either an aspartate or serine at this position (Figure 4). Further analysis of the study strain’s VP4 neutralization epitopes with corresponding VP4 neutralization epitopes of globally selected P[8]-lineage-III strains identified two amino acid differences (S146G and N113D) (Figure 4).

#### 2.3.3. Phylogenetic Analysis of VP1–VP3 and VP6

The evolutionary relationship of the VP1–VP3 and VP6 genes of the two South African study strains with a selection of global RVA strains was performed. The VP1–VP3 and VP6 genes of the two South African study strains clustered closely and displayed nearly absolute gene identities (≥99.9%) amongst each other (Figures S1–S4 in supplementary data 3 (S3)). The VP1–VP3 and VP6 genes of the two South African study strains clustered closely in sublineage composed mainly of locally circulating South African DS-1-like strains and were all found to cluster closely with cognate genes of strain RVA/Human-wt/ZAF/MRC-DPRU2344/2008/G2P[6], which was co-circulating in the population in the same year, 2008, as the study strains (Figures S1–S4 in S3). The VP1–VP3 and VP6 genes of the two study strains were closely related to cognate genes of strain RVA/Human-wt/ZAF/MRC-DPRU2344/2008/G2P[6] with nt (aa) identities ranging from ≥ 99.8–99.9% (≥99.9–100%) for VP1–VP3 and VP6 genes (S1). Phylogenetic relationship of the VP1–VP3 and VP6 genes of the atypical study strains with the cognate genes of the atypical G1P[8] strains reported in Brazil, Japan, Philippines, Thailand, Vietnam, and Malawi exhibited distinct clustering albeit belonging within the same lineage and displayed overall nt (aa) identities that ranged from ≥ 94.4–98.0% (≥ 97.1–100%) (Figure S1C–F in S3; Table S1 in S2). When VP1–VP3 and VP6 genes of the study strains were compared with selected global strains, highest genetic similarities ranging from ≥ 99.4–100% were identified with cognate genes of G3P[6] strains: RVA/Human-RVA/Human-wt/CMR/ES293/2011/G3P[6], RVA/Human-wt/TGO/MRC-DPRU2206/2009/G3G9P[6], RVA/Human-wt/BEL/F01498/2009/G3P[6], and RVA/Human-wt/UGA/MUL-13-166/2013/G3P[6] for VP1–VP3 and VP6, respectively (S1).

#### 2.3.4. Phlylogenetic Analysis of NSP1–NSP5

The NSP1–NSP5 genes of the two South African study strains were highly identical amongst each other with nt (aa) identity value of ≥99.8%, and clustered closely (Figures S5–S9). Close clustering with cognate genes of a locally circulating strain, RVA/Human-wt/ZAF/MRC-DPRU2344/2008/G2P[6], was observed for the all the NSP1–NSP5 genes, and they shared the highest nt (aa) similarities that ranged from ≥ 99.5–100% (99.4–100%) (S1). In contrast, the NSP1–NSP5 genes of the atypical study strains grouped distinctly away from cognate genes of atypical strains reported in Brazil, Japan, Philippines, Thailand, Vietnam, and Malawi and shared an overall nt (aa) similarities that ranged from ≥ 89.0–99.8% (≥94.3–100%) (Figures S5–S9 in S3; Table S1 in S2). Comparison of NSP1–NSP5 genes of the two South African study strains with corresponding selected reference strains collected globally demonstrated nt (aa) identities in the range of 98.6–100% (99.4–100%) with cognate A2, N2, T2, E2, and H2 genes of strains: RVA/Human-wt/BEL/F01498/2009/G3P[6], RVA/Human-wt/KEN/KDH1968/2014/G3P[6], RVA/Human-wt/GHA/GH018-08/2008/G8P[6], RVA/Human-wt/ZMB/MRC-DPRU1673/2009/G2P[4], and RVA/Human-wt/USA/2007769964/2007/G2P[4], respectively (S1).

### 2.4. Reassortment Analysis

**Figure 5:**
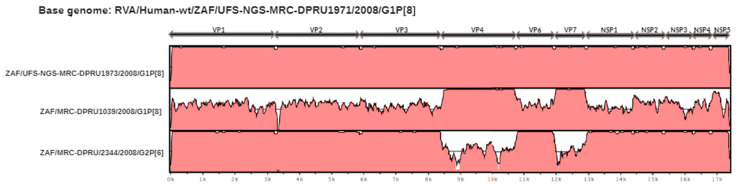
Nucleotide sequence similarities of the concatenated genome of RVA//Human-wt/ZAF/UFS-NGS-MRC-DPRU1971/2008/G1P[8] were compared with South African strains RVA//Human-wt/ZAF/UFS-NGS-MRC-DPRU1973/2008/G1P[8], RVA/Human-wt/ZAF/MRC-DPRU1039/2008/G1P[8], and RVA/Human-wt/ZAF/MRC-DPRU2344/G2P[6]. The left axis displays the strains, and rotavirus genome segment is included in the top scale. The bottom scale shows distance in kb.

The concatenated genomes of strains RVA/Human-wt/ZAF/UFS-NGS-MRC-DPRU1971/2008/G1P[8] and RVA/Human-wt/ZAF/UFS-NGS-MRC-DPRU1973/2008/G1P[8] were compared with two South African strains RVA/Human-wt/ZAF/MRC-DPRU1039/2008/G1P[8] and RVA/Human-wt/ZAF/MRC-DPRU2344/G2P[6] (Figure 5). The two atypical South African study strains shared a highly conserved backbone with all genes exhibiting > 99.8% nucleotide similarity. The VP7 and the VP4 genes of the two atypical strains shared the highest genetic similarities to RVA/Human-wt/ZAF/MRC-DPRU1039/2008/G1P[8]. However, the internal backbone genes were extremely diverse. The internal backbone genes of the atypical strains exhibited highest genetic similarity to RVA/Human-wt/ZAF/MRC-DPRU2344/G2P[6]. The results of this analysis suggest that the atypical G1P[8] strains were likely derived via reassortment events between contemporary, endemic South African strains.

## 3. Discussion

This study described the first pre-vaccine era atypical reassortant G1P[8] strains, whose outer gene segments (VP7 and VP4) expressed a Wa-like genotype, whereas the backbone genes expressed a DS-1-like genotype constellation. Analysis of the whole-genome constellation showed that genetic reassortment mechanism generated the DS-1-like G1P[8] strains. Rotavirus reassortment events are mainly facilitated by the segmentation inherent in the RV genome [2], which can generate rare or novel RV strains and hence contribute to the vast RVA diversity [22]. Wa-like and DS-1-like intergenogroup reassortment events involving G1P[8] and DS-1-like genotype constellation have been described recently in six countries: Brazil, Japan, Philippines, Thailand, Vietnam, and Malawi [23,24,25,26,27,28,29,30]. According to literature, viable atypical reassortant strains can occur under natural conditions involving Wa-like G1P[8] or G3P[8] outer capsid genes expressing DS-1-like genetic background [41,42,43,44]. This study identified two DS-1-like G1P[8] strains in the course of the ongoing AEVGI whole-genome characterization of South African RVA strains. While reported in low frequencies and limited settings in Brazil (1.6% during 2013–2017 seasons) [22], Thailand (0.4% during 2012–2014 seasons) [27], and Vietnam (14% during 2012/2013 season) [28,29], 31–62% of these DS-1-like G1P[8] strains accounted for RVA positive strains circulating across selected regions in Japan [26], and 40% of randomly sampled post-vaccine samples were reported in Malawi [24]. Such atypical reassortant strains have the potential to predominate in circulation. G1P[4] strains suggested to have emerged from intergenogroup reassortment events accounted for 41% of RVA strains circulating in the peak months of the 2001 RVA season in Detroit, USA [45], whereas a surge in G3P[4] strains also presumed to have emerged from intergenogroup reassortment events were detected in Brazil at 36% [46] and in Ghana at 64% [47]. The two South African study strains were identified during the pre-RVA vaccination period in South Africa in contrast to the previously reported atypical strains found during the post-RVA vaccination period. This implies that the reassortment events that led to the emergence of the South African atypical G1P[8] strains may not necessarily be driven by vaccine-induced selective pressure but by natural evolutionary processes of RVA genome.

In order to identify the ancestral origin of these G1P[8] strains, assessment of whole-gene sequences and phylogenetic analysis showed that the outer capsid genes, VP7 and VP4, of strains RVA/Human-wt/ZAF/UFS-NGS-MRC-DPRU1971/2008/G1P[8] and RVA/Human-wt/ZAF/UFS-NGS-MRC-DPRU1973/2008/G1P[8] were 99.6–99.9% (99.1–100%) identical with South African strain RVA/Human-wt/ZAF/MRC-DPRU1039/2008/G1P[8] and clustered together in the same clade within the same. For the internal genes, the highest nucleotide identities were identified with cognate genes of another South African strain, RVA/Human-wt/ZAF/MRC-DPRU2344/2008/G2P[6], detected in the 2008 RVA season. Put together, it is probable that a locally circulating G2P[6] strain such as RVA/Human-wt/ZAF/MRC-DPRU2344/2008/G2P[6] with a DS-1-like backbone derived the VP7 and the VP4 genes from a locally co-circulating G1P[8] strain such as RVA/Human-wt/ZAF/MRC-DPRU1039/2008/G1P[8], generating the double-gene reassortants. Consequently, the results obtained in this study indicate that the two atypical South African G1P[8] strains were generated locally through genetic reassortment events. The generation of these reassortant double-gene strains tend to be independent of the events in which Brazilian, Japanese, Thai, Vietnamese, and Malawian DS-1-like G1P[8] strains were generated. The nine internal genes of the South African DS-1-like G1P[8] strains always clustered together with cognate genes of a locally circulating G2P[6] strain distinctly away from the cluster comprising Japanese, Thai, Philippines, and Malawi DS-1-like G1P[8] strains. Therefore, the South African DS-1-like G1P[8] strains emerged clonally from independent events, a phenomenon observed for the Malawian [24] and the Vietnamese [30] DS-1-like G1P[8] strains. In contrast, Brazilian, Japanese, and Thai G1P[8] DS-1-like strains were established to have been derived from a common ancestor [23,29].

Vaccine escape mutants can result due to mutations occurring in well-known VP7 neutralization epitope regions [48]. Host–antigen binding interactions involving human G1 strains are significantly impacted by mutations occurring at positions 94, 97, 147, and 291 [48]. The identified N94S substitution involving the substitution of asparagine (N) with a serine(S), which are both polar non-charged amino acid residues [49], may not significantly alter the overall morphology of the protein surface. However, since asparagine is usually N-glycosylated, there is a likely loss of glycosylation site, which could have a wide-ranging impact on the immunogenicity of the 7-1a epitope [50]. The D97E amino acid substitution involving polar negatively charged residues, aspartate (D) and glutamate (E), is likely to be a silent nucleotide change [49]. Similarly, a K291R substitution involving lysine (K), an amphipathic polar amino acid, and arginine (R), a positively charged amino acid, is unlikely to have a far-reaching structural effect on the VP7 protein surfaces. However, M217T substitution was identified resulting in substitution of methionine, a non-polar residue to threonine, a polar amino acid residue, which could likely result in significant changes in biochemical properties of VP7 [49]. This M217T substitution was also present in earlier strains as well as some post-vaccine strains that were included for analysis, and the role it plays in driving epidemiological fitness of G1 strains is not fully resolved. In the host cell, trypsin-like proteases cleave the VP4 spike protein into two structural domains (VP8* and VP5*) [2]. Four surface-exposed antigenic epitopes (8-1 to 8-4) have been described in the VP8* region, while five antigenic epitopes (5-1 to 5-5) in the VP5* region have been documented [40]. The amino acid changes E150D, N195G, S125N, and N135D that were observed relative to the vaccine strains were conservative. However, a S131R substitution that resulted in a change in polarity might play a role in escape of host immunity [49]. Another amino acid substitution R131S resulting in a change in charge from positively charged amino acid to non-charged amino acid that was identified when comparison was made against globally selected lineage-III VP4 strains might impact vaccine escape effect [49].

## 4. Materials and Methods

### 4.1. Ethics Approval

The study was approved under ethics number UFS-HSD2018/0510/3107 by the Health Sciences Research Ethics Committee (HSREC) of the University of Free State, Bloemfontein, South Africa. The patient identities and demographics were de-linked from their unique laboratory identifiers to ensure confidentiality.

### 4.2. Sample Collection

Rotavirus positive stool samples from children under five years of age treated for gastroenteritis at Dr. George Mukhari Hospital, Pretoria North, South Africa and conventionally genotyped as G1P[8] were sourced from archival storage (2002 to 2017) of South Africa Medical Research Council—Diarrheal Pathogens Research Unit (MRC-DPRU), a WHO Rotavirus Regional Reference Laboratory (WHO-RRL) in Pretoria, South Africa. The two stool samples that were later genotyped as DS-1-like G1P[8] strains were collected from 6-month female and 12-month male children on 15 and 16 May 2008, respectively, from Soshanguve, Pretoria.

### 4.3. Extraction and Purification of Double-Stranded RNA

The extraction of RV ds-RNA was conducted by utilizing a previously described method [51], albeit with modifications (UFS-NGS unit extraction SOP). Briefly, a pea size (~100 mg) sample of stool was added to 200 µL of phosphate-buffered saline (PBS) solution, pH 7.2 (Sigma-Aldrich^®^, St Louis, MO, USA). The solution was mixed by pulse-vortexing for five seconds. A 1 mL volume of TRI-Reagent^®^-LS (Molecular Research Center, Inc, Cincinnati, OH, USA) was added and let to stand for five minutes. Phase separation was achieved by addition of 270 µL of chloroform (Sigma-Aldrich^®^, St Louis, MO, USA). Afterward, centrifugation for 13,000 revolutions per minute (RPM) was performed for 20 min at 4 °C in a temperature-controlled microcentrifuge (Eppendorf microcentrifuge 5427R, Hamburg, Germany). A volume of 1 mL isopropanol (Sigma-Aldrich^®^, St Louis, MO, USA) was added to the supernatant, and centrifugation was performed at 13,000 RPM for 30 min at room temperature. The supernatant was poured off, and the tubes were let to dry for 10 min, after which 95 µL of elution buffer (EB) from the MinElute Gel extraction kit (Qiagen, Hilden, Germany) was added. A 30 µL volume of of 8M LiCl2 (Sigma, St. Louis, MO, USA) was added, and the solution was precipitated for 16 h at 4 °C in a water bath in a Tupperware box. The MinElute gel extraction kit (Qiagen, Hilden, Germany) was used to purify the extracted RNA according to manufacturer’s instructions, and 1% 0.5 X TBE agarose gel stained with Pronasafe (Condalab, UK) electrophoresis was used to verify the integrity and the enrichment of dsRNA, which was visualized on a G:Box Syngene UV transilluminator (Syngene, Cambridge, UK).

### 4.4. Synthesis and Purification of Complementary DNA (cDNA)

The Maxima H Minus Double-Stranded cDNA Synthesis Kit (Thermo Fischer Scientific, Waltham, MA) was utilized to synthesize cDNA from the extracted viral RNA. Briefly, denaturation at 95 °C for 5 min of the extracted RNA was performed followed by addition of 1 µL of 100 µM Random Hexamer primer. Incubation was performed in a thermocycler at 65 °C for five minutes. The First-Strand Reaction mix (5 µL) and the First Strand Enzyme Mix (1 µL) were added, and the solution was incubated at 25 °C for 10 min followed by 2 h at 50 °C, and then the reaction was terminated by heating at 85 °C for 5 min. A volume of 55 µL of nuclease-free water, 20 µL of 5X Second Strand Reaction Mix, and 5 µL of Second Strand Reaction Mix was then added. The solution was then incubated at 16 °C for 60 min, after which the reaction was stopped by adding 6 µL 0.5M EDTA. A volume of 10 µL RNAse I was then added, and the synthesized cDNA was incubated for five minutes at room temperature. Subsequently, the MSB^®^ Spin PCRapace (Stratec) Purification Kit was used to purify the synthesized cDNA.

### 4.5. DNA Library Preparation and Whole-Genome Sequencing

The Nextera^®^ XT DNA Library Preparation Kit (Illumina, San Diego, California, US) was utilized to prepare DNA libraries by following manufacturer’s instructions. Briefly, the genomic DNA was tagmented by using the Nextera^®^ transposome enzyme, and the tagmented DNA was subsequently amplified using a limited-cycle PCR program. The DNA libraries were cleaned-up using AMPure XP magnetic beads (Beckman Coulter, Pasadena, CA, USA) and 80% freshly prepared ethanol. The quantity of the DNA was determined using Qubit 2.0 fluorometer (Invitrogen, Carlsbad, CA, USA), and the quality of the libraries and the fragment sizes was assessed using Agilent 2100 BioAnalyzer^®^ (Agilent Technologies, Waldbronn, Germany) by following the manufacturer’s specified protocol. The Illumina MiSeq^®^ sequencer (Illumina, San Diego, CA, USA) was utilized to perform paired-end nucleotide sequencing (301 × 2) for 600 cycles by using a MiSeq Reagent Kit v3 at the University of the Free State-Next Generation Sequencing (UFS-NGS) Unit, Bloemfontein, South Africa.

### 4.6. Genome Assembly

Geneious Prime^®^ software, version 2019.1.1 (Biomatters, https://www.geneious.com/; [52]) was used for genome assembly. Briefly, for use with the reference mapping tools integrated in Geneious Prime version 2019.1.1, the default medium sensitivity parameter was selected to generate contigs from the FASTQ files data generated by the Illumina MiSeq^®^ instrument. Complementary RV genome assembly was also performed using an in-house genome assembly pipeline and CLC Genomics Workbench 12 (https://www.qiagenbioinformatics.com/).

### 4.7. Determination of Rotavirus Whole-Genotype Constellations

The genotype of each gene segment was determined using Rota C, v 2.0 [13], an online server for genotyping RVA strains. This was used to generate the full genotype constellations for each RV strain.

### 4.8. Phylogenetic Analyses

Complete sequences for each gene segment were aligned and sequence comparisons performed as described previously [53,54,55]. Multiple sequence alignments were implemented utilizing the MUSCLE package in Molecular Evolutionary Genetics Analysis (MEGA) 6 software ([56]; http://www.megasoftware.net/). Upon alignment, the DNA Model Test program in MEGA 6 was used to determine the evolutionary model that best fits each gene sequence datasets. The models identified as best fitting with the sequence data for the indicated genes using the Corrected Akaike Information Criterion (AICc) were as follows: GTR+G+I (VP7, VP4, VP6, VP1, VP2, VP3, NSP1, NSP2, NSP3) and HKY+G+I (NSP4 and NSP5). These models were utilized in maximum-likelihood trees’ construction using MEGA 6 with 1000 bootstrap replicates to estimate branch support. Genetic distance matrices were prepared using the *p*-distance algorithm of MEGA 6 software [56]. In addition to the two whole-genome sequences of the strains in this study, other cognate sequences were acquired from GenBank ([57]; http://www.ncbi.nlm.gov/genbank). Further phylogenetic analysis by geographical regions Africa (Eastern Africa, Southern Africa, and West Africa), Asia, Americas, Europe, and Oceania was also performed. mVISTA software was used to visualize the comparative sequence similarities of concatenated whole-genome of genetically related strains [58].

## 5. Conclusions

Whole-gene analyses showed that the South African DS-1-like G1P[8] strains were generated involving locally circulating G2P[6] strains by acquiring the VP7 and the VP4 outer capsid proteins of locally co-circulating G1P[8] strains. Similar to their pre-vaccine era detection in Vietnam and Philippines, the identification of these atypical DS-1-like G1P[8] strains during the pre-vaccine period in South Africa, as opposed to their detection during post-vaccination era in selected settings in Brazil (Sao Paulo and Goias in 2013), Japan (Okayama, Aichi, Akita, Kyoto, and Osaka Prefectures in 2012), Thailand (Phetchabun and Sukhothai in 2013), and Malawi (Blantyre in 2013/2014), suggests that they originated from natural evolutionary processes of RVA genome. Whole-genome surveillance of RVA genotypes is imperative to understand the occurrence rate, the mechanisms that drive emergence of such atypical strains, and their epidemiological fitness as well as to assess the effect of vaccine selective pressure in shaping the antigenic landscape of RVA strains.

## Figures and Tables

**Table 1:** Whole genotype constellations of the South African DS-1-like G1P[8] rotavirus strains.

Gene Segment	VP7	VP4	VP6	VP1	VP2	VP3	NSP1	NSP2	NSP3	NSP4	NSP5
**Base pair size for full length sequences**	1062	2359	1355	3302	2684	2591	1566	1059	1066	751	810
**Base pair size for the complete study strain ORF**	978	2325	1191	3264	2637	2505	1458	951	939	525	600
**RVA/Human-wt/ZAF/UFS-NGS-MRC-DPRU1971/2008/G1P[8]**	G1	P[8]	I2	R2	C2	M2	A2	N2	T2	E2	H2
**RVA/Human-wt/ZAF/UFS-NGS-MRC-DPRU1973/2008/G1P[8]**	G1	P[8]	I2	R2	C2	M2	A2	N2	T2	E2	H2

Color codes indicate genogroup attribution. Green color represents the genotype associated with the Wa-like genogroup, while red color represents the genotype belonging to the DS-1-like genogroup. The nomenclature of the RV strains indicates RV group, species where the strain was isolated, name of the country where the strain was originally isolated, common name, year of isolation, and genotypes for genome segments four and nine as proposed by the Rotavirus Classification Working Group (RCWG) [12]. ORF = open reading frame.

## Supplementary Materials

The following are available online at https://www.mdpi.com/2076-0817/9/5/391/s1, Supplementary data 1 (S1): Identity matrices analysis for VP1, VP2, VP3, VP4, VP6, NSP1, NSP2, NSP3, NSP5 and NSP5 nucleotide and deduced amino acid identities among strains calculated by distance matrices using P-distance algorithm in MEGA 6. Supplementary data 2 (S2): Homology analysis summary containing Table S1 and Table S2. Supplementary data 3 (S3): Additional phylograms containing Figure S1:VP1, S2:VP2, S3:VP3, S4:VP6, S5:NSP1, S6:NSP2, S7:NSP3, S8:NSP4 and S9:NSP5.
